# A nursing report on a corneal contact lens wearer receiving keratoplasty due to corneal ulcer and perforation caused by *Pythium insidiosum* infection: A case report

**DOI:** 10.1097/MD.0000000000037663

**Published:** 2024-04-05

**Authors:** Shui-hua Lu, Dan-ni Qiao, Pei-fang Dong

**Affiliations:** a Department of Nursing, The Second Affiliated Hospital of Zhejiang University School of Medicine, Hangzhou, China; b Department of Eye Center, The Second Affiliated Hospital of Zhejiang University School of Medicine, Hangzhou, China.

**Keywords:** Corneal contact lens, corneal ulcer, keratoplasty, nursing, *Pythium insidiosum*

## Abstract

**Background:**

To report the nursing experience of a case of corneal contact lens wearer receiving the 2nd keratoplasty due to corneal ulcer and perforation caused by *Pythium insidiosum* infection.

**Methods:**

A 30-year-old female patient had blurred vision after deep anterior lamellar keratoplasty for a right corneal ulcer. At the 5th week, the right eye appeared the symptoms, such as redness and pain. The anterior segment photography was performed on the eye, and the result showed that the epithelium was missing in the right eye lesion area, and a large number of longitudinal and transversal streaks were visible from the epithelium to the stroma, with fungus filaments to be discharged. Upon macro-genome sequencing of the corneal secretion, a *P. insidiosum* infection was observed. Then, the patient underwent the keratoplasty, and 3 weeks later, the corneal implant showed a tendency to dissolve, the sutures were partially loosened, and the eye was almost blind. Subsequently, the patient was admitted to our hospital and subject to the 2nd penetrating keratoplasty of the right eye (allograft). After surgery, linezolid and azithromycin injections were given through intravenous drip and local drip of the eye for anti-inflammation, and tacrolimus eye drops for antirejection.

**Results:**

Postoperatively, the patient showed signs of recovery with slight corneal edema and visible pupil, leading to discharge with improved vision. The corneal implant was normal 1 week after surgery and the vision of the right eye was hand move/before eye at the 6th month of follow-up. Continuous care and removal of sutures 3 months post-surgery contributed to a successful outcome, with the patient achieving hand motion vision 6 months after the procedure.

**Conclusion:**

Corneal ulcer caused by *P. insidiosum* infection not only needs timely and effective keratoplasty intervention, but also requires perfect nursing measures.

## 1. Introduction

Wearing corneal contact lenses is a convenient way to correct ametropia. Notably, compared with frame glasses, corneal contact lenses have many advantages. However, infectious keratitis caused by the irregular use of corneal contact lenses is one of the most serious complications, and its incidence is 0.03% to 0.05%.^[1,2]^ Even, infectious keratitis can leads to permanent loss of vision eventually by inducing corneal perforation and endophthalmitis.^[3]^ *Pseudomonas aeruginosa* and *Acanthamoeba* infections are the most common microbial infection sources, accounting for 38% and 50% of infectious keratitis, respectively, among which washing the contact lenses with tap water is one of the high-risk factors of infecting pathogens.^[4–6]^ *Pythium insidiosum* is an aquatic oomycete, which mostly exists in Southeast Asia, and *Pythium* keratitis in this area accounts for 5.9% to 6.2% of the keratitis incidence.^[7]^ Owing to its virulence, *P. insidiosum* is not sensitive to conventional antibacterial treatment. If the early infection is not controlled, *P. insidiosum* may result in corneal ulcer perforation eventually and even lead to eyeball removal. At present, keratoplasty is the only treatment for such patients. However, even after keratoplasty, there is still the possibility of *P. insidiosum* residue in the stroma, which not only leads to a high-risk of postoperative recurrence and a very poor prognosis but also brings great challenges to nursing work. In December 2022, our department admitted a patient with corneal ulcer and perforation caused by *P. insidiosum* infection due to improper care of contact lens. This patient received the 2nd keratoplasty in our department. After careful perioperative nursing, the patient got better, was discharged and had good prognosis upon surgery. The report was shown as follows.

## 2. Case presentation

The patient was a 30-year-old female admitted to the hospital on December 15, 2022 for 5 weeks of blurred vision after deep anterior lamellar keratoplasty due to corneal ulcer in the right eye. On September 19, 2022, the symptoms of redness and pain in the right eye were obvious, and the patient was sent to outpatient department. The anterior segment photography of the eye showed that the epithelial layer in the right eye lesion area was missing, a large number of criss-crossing cord-like light strips were visible from the epithelium to the stroma layer, and the fungus filaments remained to be discharged. The outpatient department gave oral antifungal therapy for treatment. The bacterial cultures of specimens were collected by corneal scraping. On October 3, the culture result showed no fungus or virus growth, and on October 6, the results of macro-genome sequencing of the corneal secretion showed *P. insidiosum* infection. After routine antifungal and anti-infection treatment, the disease was not controlled, but progressed rapidly. On November 9, deep anterior lamellar keratoplasty of the right eye (allograft) was performed under local anesthesia. After surgery, azithromycin and levofloxacin eye drops were given for anti-infection, and tacrolimus eye drops for antirejection. On November 23, due to the opacity of corneal implant and exudation of anterior chamber in the right eye, irrigation of right anterior chamber + re-suture of the implant + macro-genome sequencing of the secretions was performed under local anesthesia. Upon surgery, anti-infection treatment was carried out through intravenous drip of linezolid and glucose injection. Three weeks after the irrigation of the right anterior chamber, the corneal implant showed a dissolution trend, the suture was loosened in part, and the eye almost could not work. On December 15, the patient was admitted to the hospital for a 2nd keratoplasty.

**Figure 1.**
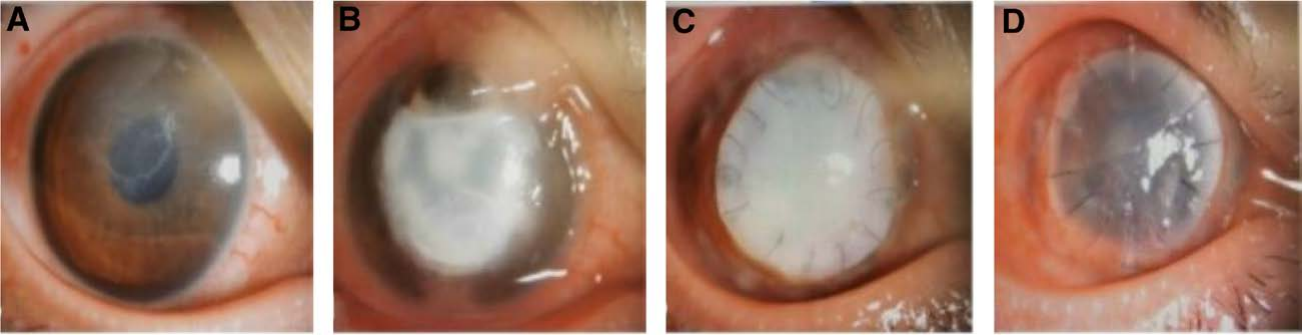
Corneal appearance of patients at different times before and after keratoplasty: (A) Corneal infiltration at the initial stage of treatment; (B) Corneal ulcer after 2 months of treatment; (C) Corneal dissolution and suture loosening occurred 1 month after lamellar keratoplasty; (D) Normal corneal implant film 1 week after penetrating keratoplasty.

The physical examination results were shown as follows: the body temperature (36.8°C), pulse (78 times/minute), breathing (18 times/minute), and blood pressure (136/78 mm Hg). Specialist examination: the right naked eye had no light perception, and the corrected vision in the left eye was 1.0; intraocular pressure in the right eye was 16.5 mm Hg, the left eye was 18.1 mm Hg; both external eyes were negative, the conjunctiva of the right eye was congested, the eye position was normal, the corneal implant was preserved and cloudy, the temporal side was open, the suture was partially loose, and the rest structures were invisible. The left conjunctiva was free of congestion, the cornea was transparent and the anterior chamber was clear and deep. The patient was admitted to the hospital for diagnosis of the right corneal implant dissolution and right keratitis. Preoperative preparation was improved immediately after admission, and penetrating keratoplasty of right eye (allograft) was performed under general anesthesia on December 16. After surgery, linezolid and azithromycin injection (intravenous drip and local drip of the eye) were employed for anti-inflammation, and tacrolimus eye drops for antirejection. On the 2nd day after surgery, the corneal implant was slightly edematous, the pupil was visible, the suture was preserved, and the patient was discharged successfully. The corneal implant was normal 1 week after surgery, and the visual acuity of the right eye was hand move/before eye after 6 months of follow-up (Fig. 1).

## 3. Nursing

### 3.1. Creative cognitive reappraisal for psychological nursing

The patient is a young white-collar worker who needed a 2nd keratoplasty due to the infection of cornea with rare bacteria caused by washing the corneal contact lenses directly with tap water. Through communication, nurses learned that the patient was guilty, absent from correct cognition, and not confident to the success rate of keratoplasty. In view of the above negative emotions, the Self-Rating Depression Scale (SDS) was used for evaluation, and the score was 51, which indicated mild depression.

The creative cognitive reappraisal was applied to improve the negative emotions of this patient. Specifically, nurses: used warm language and positive attitude to calm the patient and stabilize her emotions during admission evaluation; patiently and professionally answered questions from the patient and her families during hospitalization, and gained the trust of patient; introduced the purpose, method and successful cases of surgery at home and abroad to the patient, so as to help her build up the courage and confidence to overcome the disease; selected 20 negative pictures from the international affective picture system and wrote a creative cognitive evaluation. On the 1st day of admission, the creative cognitive reappraisal was performed with the informed consent of the patient, so that the patient could understand the pictures from multiple angles according to the text description as much as possible and tried her best to imagine that the event happened to herself.

The patient thought that “the current situation was not as bad as imagined” and “learning lessons could help protect the healthy left eye from infection,” and actively cooperated with treatment and nursing during hospitalization. On the day of discharge, the patient’s SDS score was 42, and the result was negative.

## 4. Intraocular pressure management in perioperative period of keratoplasty

### 4.1. Observation and nursing of intraocular pressure

The patient had hypopyon, large range of corneal lesions, corneal ulcer, and perforation before surgery, and all these were high-risk factors for ocular hypertension. A good preoperative intraocular pressure management is an important link to reduce the risk of surgery. Therefore, the nurse in charge: measured the intraocular pressure immediately after admission of the patient and taught the patient to accurately describe the property and degree of pain referring to the pain/facial digital score table posted on the ward wall; used levofloxacin eye drops to reduce the inflammatory response of the eye; adopted mannitol, a reserve medicine, to reduce intraocular pressure when the patient suffered from high intraocular pressure. The intraocular pressure of the patient’s right eye was 16.1–18.8 mm Hg from admission to surgery, and there was no preoperative high intraocular pressure.

Ophthalmology nurses fully recognized the risk of postoperative ocular hypertension in this patient, and formulated the targeted preventive nursing measures as follows: early ocular hypertension occurred frequently within 24 hours after surgery, so the patient was asked to report adverse reactions, such as eye swelling, eye pain, headache, nausea, and vomiting in time; besides, the intraocular pressure and visual recovery were measured twice a day, and the recovery of the implant and the implant bed was observed by a slit lamp biomicroscope. The patient was told to eat fiber-rich vegetables and fruits after surgery, and avoid eating greasy and fried foods; if the constipation occurred, forcible defecation was forbidden to avoid the rupture of the implant and the implant bed. Excessive head movements that might increase intraocular pressure were avoided. On the day of surgery, the affected eye was covered with an eye patch, the patient felt slightly swollen, and the pain score was 2 points. The intraocular pressure of the right eye was 15.8–19.5 mm Hg on the next day after surgery.

### 4.2. Selection of the appropriate intraocular pressure measurement tool

A rebound tonometer was selected to avoid the risk of corneal injury and improve the accuracy of intraocular pressure measurement after keratoplasty. To be specific, the measurement position and angle are particularly important for the accuracy of measurement results, so the patient was asked to take the correct sitting position, relax herself and look straight ahead before starting the measurement; the sterile probe was changed for measurement with the rebound tonometer each time, and the length of the support rod was adjusted until the pressure measuring tip was 4–8 mm away from the corneal apex; the measurement key was continuously pressed after the reading returned to zero, and if the measurement was successful obtained for 6 times in a row, the measurement average was calculated; for the patient after keratoplasty, the tonometer was wiped with alcohol wipes before and after intraocular pressure measurement. During hospitalization, the intraocular pressure measurement of this patient was performed by bedside, with good cooperation and without operational corneal injury.

## 5. Medication nursing of linezolid

The patient was given the linezolid injection (0.6 g) through intravenous drip every 12 hours for 7 days before the 2nd keratoplasty; besides, linezolid was prepared as the eye drops and then dripped into the right eye 4 times a day. The nursing measures during medication were listed as follows: the necessity and risks of applying linezolid were introduced to the patient and her families, and the possible adverse reactions during medication needed to inform the medical staff; the nurse in charge was responsible for assessing whether there was a bleeding point, abdominal pain, change of stool color and shape, and stomach upset in the patient; the blood routine indexes as well as liver and kidney functions were checked after 3 days of systemic medication; the patient with a wide range of medications and reduced activities was prone to flora imbalance; in addition to monitoring the patient’s body temperature, the nurse in charge was also responsible for evaluating whether the perineal skin existed the signs of secondary infection and assisting patients in cleaning perineum and urinary tract every day; linezolid was injected intravenously within 30–120 minutes, and to avoid chemical compatibility between drugs, the infusion set was washed with 0.9% sodium chloride injection before and after infusion. The patient did not suffer from any adverse drug reactions during hospitalization.

## 6. Nursing of preventing complications

### 6.1. Cross-infection of the contralateral eye

The patient’s right cornea was infected with *P. insidiosum* because of wearing the lens after cleaning it with tap water. Finally, the patient developed corneal perforation and received keratoplasty. Therefore, it was urgent to prevent cross-infection of the contralateral eye: anti-infection treatment with levofloxacin and linezolid eye drops during perioperative period; the patient was placed in a bed by the window to minimize the chance of contact with other people; a disposable surgical bag was applied in keratoplasty to prevent cross-infection; the eye drops were bagged separately, the hands were washed with running water before and after contact, and the hand hygiene was strictly implemented; under the incomplete control of inflammation, the contralateral eye no longer wore contact lenses. The surgical eye infection was controlled after surgery, and no contralateral eye infection occurred during hospitalization.

### 6.2. Cavernous sinus thrombophlebitis

The patient was seriously infected by *P. insidiosum*, and only 2 months, the redness of eyes was developed to corneal perforation, so it was needed to closely observe the condition to avoid serious life-threatening complications. Preventive measures were displayed as follows: the responsible nurse measured the patient’s temperature 3 times a day, and once every 4 hours when the condition was abnormal; the abnormal manifestations, such as eye swelling, drooping eyelids, limited eye movement, and diplopia in each shift were evaluated; cavernous sinus thrombosis was easy to cause brain edema, so the mental state of patients, the presence of headache, and the changes in consciousness and pupils were observed in each shift; an emergency plan was developed for the occurrence of cavernous sinus thrombophlebitis to reduce intracranial pressure and relieve brain edema. The patient’s vital signs were stable and there was no cavernous sinus thrombophlebitis during hospitalization.

## 7. Continuous nursing

Long-term attention needed to be paid to medication and follow-up after penetrating keratoplasty. Reasonable discharge preparation service could not only ensure the patient’s smooth transition from hospital to family, but also improve the prognosis of surgery.

### 7.1. Observation and nursing of implant

Due to corneal donor, postoperative immune rejection, chronic failure of corneal implant, recurrence of primary disease, and other reasons, the function of corneal endothelial cells could not be compensated or corneal stroma was turbid. Therefore, the symptomatic treatment or the 2nd keratoplasty needed to be carried out according to the actual situation of the patient. The measures for the patient were presented as follows: the medical team evaluated the patient and her family, and a discharge plan was initially developed; the responsible nurse used the real pictures of outpatient treatment, including normal and abnormal implants after keratoplasty, to teach the patient and her families to observe the implant and distinguish the implant abnormalities by themselves; on the 1st day before discharge, the nurses assessed the patient and her families’ knowledge; on the day of discharge, the patient and her families were taught to fill in the electronic observation record form, the abnormal implant manifestations of the patient’s right eye, including eye redness, eye pain, secretion, whether the color of the implant was white or not, whether the suture was loose, were recorded, and the clear photos were taken with the mobile phone and shared in the WeChat communication group; the patient was suggested to choose Internet plus online consultation; before the suture was removed, anti-inflammatory and antirejection eye drops were still needed to strengthen eye hygiene and prevent dirty water from entering the eyes; within 1 year after keratoplasty, the corneal nerve tissue was not completely repaired, the cornea was insensitive, rubbing the eye was not allowed, and the trauma was prevented.

### 7.2. Timing of corneal suture removal

The timing of suture removal was informed according to the patient’s individual situation: the suture removal began gradually 1 month after routine surgery, and the sutures were removed within 3–6 months; if the suture loosened within 1 month after surgery, the patient needed to be visited by a doctor immediately, and the doctor would decide whether it was necessary to sew again after evaluation; once the corneal implant was found to be white, the periphery of the cornea was rolled up and the infection occurred due to loose corneal suture, then the patient needed to be visited by a doctor immediately. Two weeks after the 2nd penetrating keratoplasty, the patient had a loose suture of the right cornea and a slight opacity of the implant. Immediately after seeing a doctor, the loose suture was removed, the right cornea was sutured again, and the eye drops continued to be given for anti-infection. Three months after surgery, the corneal sutures in the right eye were all removed.

## 8. Discussion and conclusions

Corneal ulcer caused by *P. insidiosum* infection after wearing contact lens is rare. The 2nd penetrating keratoplasty was performed in this case because the deep lamellar keratoplasty failed. Once the *P. insidiosum* infection source spreads into the eye, the eyeball may be subject to the enucleation. Furthermore, due to the refractory property and high blindness rate, infectious corneal ulcer can induce the gradual accumulation of negative emotions. These negative emotions may lead to depression, self-abandonment, and loss of confidence in treatment and life, if the patients cannot guide and transfer the pressure well and correctly face up to the treatment and prognosis of the disease.^[8]^ Therefore, patients’ negative emotions need to be identified as soon as possible and effectively intervened. The results of SDS evaluation revealed that the patient had mild depression. Based on this, we applied creative cognitive reappraisal to try to improve the patient’s negative emotions. Creative cognitive reappraisal refers to a highly creative, novel and unique interpretation adaptable to current emotional stimuli. When individuals combine this interpretation framework with the stimulus situation in the process of emotional regulation, they will be suddenly enlightened.^[9]^ Creative cognitive reappraisal can turn negative emotions into positive ones, which is an efficient and feasible emotion regulation strategy.^[9,10]^ After effective nursing and introduction of successful cases by nurses, the patient’s negative emotions were obviously improved. This is beneficial to our subsequent surgical treatment and postoperative nursing work.

Studies have stated that postoperative secondary glaucoma is the most common complication of penetrating keratoplasty and an important cause of endothelial function decompensation or immune rejection of corneal implants. Postoperative viscoelastic residue, inflammatory stimulation, hyphema, and implicated tissue can block trabecular meshwork and lead to severe postoperative ocular hypertension. Residual viscoelastic agent in anterior chamber is the most common cause of transient ocular hypertension after surgery. The literature has reported that the incidence of early secondary glaucoma after penetrating keratoplasty is 9% to 31%, and the incidence of late secondary glaucoma is 18% to 35%,^[11]^ which first appears at 3–24 hours after surgery.^[12,13]^ Therefore, in the case that the ophthalmologist nurses predicted the postoperative risk of high intraocular pressure, the targeted preventive nursing measures were formulated, which could effectively prevent complications and promote the patient’s rehabilitation as soon as possible.

Linezolid is an inhibitor of bacterial protein synthesis used to treat infections caused by Gram-positive (G+) cocci. Antibiotics such as linezolid and azithromycin, which can inhibit the synthesis of protein, are more effective in treating *P. insidiosum* infection than antifungal therapy.^[14,15]^ However, adverse drug reactions caused by linezolid involving the blood system was the highest (53.62%), manifested as thrombocytopenia, anemia, pancytopenia, and leukopenia. Therefore, to reduce the adverse reactions caused by drugs, in addition to careful medication, we also explained the possible adverse reactions of drugs, so as to avoid patients’ worries, fears and panic when side effects occurred.

Cavernous sinus thrombophlebitis is a fatal complication of *Pythium* corneal infection.^[16]^ *P. insidiosum* is highly toxic, which may lead to serious ocular inflammation due to the inflammation progressing into the eye, involving the orbit and leading to early panophthalmia. Through venous reflux, inflammation complicates with cavernous sinus thrombophlebitis, which is often accompanied by sepsis and meningitis. Moreover, the clinical misdiagnosis rate of this complication is over 50% and the mortality rate is particularly high.^[16]^ Therefore, when observing the condition, the doctor needs to identify ocular hypertension as early as possible to prevent secondary glaucoma, be alert to the occurrence of life-threatening cavernous sinus thrombophlebitis, and strengthen the observation of drug efficacy and adverse reactions. In this study, we also formulated detailed nursing measures to prevent the patient from postoperative cavernous sinus thrombophlebitis after discharge.

All in all, after our surgical treatment and detailed nursing measures, the patient has basically recovered without postoperative complications and adverse drug reactions. However, it is still necessary to continue to pay attention to postoperative implant opacity, corneal sutures and follow-up to improve the success rate of keratoplasty.

## Author contributions

**Conceptualization:** Shui-hua Lu.

**Investigation:** Dan-ni Qiao.

**Writing—original draft:** Pei-fang Dong.

**Writing—review & editing:** Pei-fang Dong.
